# Associations of complement factor B and complement component 2 genotypes with subtypes of polypoidal choroidal vasculopathy

**DOI:** 10.1186/1471-2415-14-83

**Published:** 2014-06-25

**Authors:** Koji Tanaka, Tomohiro Nakayama, Ryusaburo Mori, Naoyuki Sato, Akiyuki Kawamura, Mitsuko Yuzawa

**Affiliations:** 1 Department of Ophthalmology, Nihon University School of Medicine, 1-8-13 Kandasurugadai, Chiyoda-ku, Tokyo 101-8309, Japan; 2 Department of Pathology and Microbiology, Nihon University School of Medicine, Tokyo, Japan

**Keywords:** Subtypes of PCV, C2, CFB, Genetic variants

## Abstract

**Background:**

We previously reported on subtypes of polypoidal choroidal vasculopathy (PCV), and categorized PCV as polypoidal choroidal neovascularization (CNV) and typical PCV. The aim of this study was to clarify whether complement component 2 (*C2*) and complement factor B (*CFB*) genotypes are associated with subtypes of polypoidal choroidal vasculopathy, such as polypoidal CNV and typical PCV.

**Methods:**

First, we categorized 677 patients into typical age-related macular degeneration (tAMD; 250 patients), PCV (376) and retinal angiomatous proliferation (RAP; 51). Second, we categorized 282 patients with PCV as having polypoidal CNV (84 patients) or typical PCV (198) based on indocyanine green angiographic findings. In total, 274 subjects without AMD, such as PCV and CNV, served as controls. A SNP (rs547154) in the *C2* gene and three SNPs (rs541862, rs2072633, rs4151667) in the *CFB* gene were genotyped, and case–control studies were performed in subjects with these PCV subtypes.

**Results:**

In tAMD, no SNPs were associated with allele distributions. In PCV, rs547154 and rs2072633 were associated with allele distributions. RAP was only associated with rs2072633. After logistic regression analysis with adjustment for confounding factors, tAMD, PCV and RAP were found to be associated with rs2072633.

As to PCV subtypes, there were significant differences in the distributions of rs547154, rs541862 and rs2072633 in the case–control studies for polypoidal CNV, but not between the typical PCV and control groups. Logistic regression analysis with adjustment for confounding factors showed the distributions of rs547154, rs541862 and rs2072633 to differ significantly between the controls and polypoidal CNV cases and that these SNPs were protective. The A/A genotype of rs2072633 was significantly more common in the polypoidal CNV than in the typical PCV group (p = 0.03), even with adjustment for polyp number and greatest linear dimension.

**Conclusions:**

PCV might be genetically divisible into polypoidal CNV and typical PCV. The C2 and CFB gene variants were shown to be associated with polypoidal CNV. Typical PCV was not associated with variants in these genes.

## Background

Age-related macular degeneration (AMD) is a leading cause of blindness in Western countries and its prevalence is increasing in Japan [1]. AMD is thought to be a heterogeneous multifactorial disease associated with several environmental factors and genetic variants. Hypertension [2] and cigarette smoking [3] are closely related to the development of AMD. Identification of AMD susceptibility genes might increase our ability to predict the risk of developing this disease. Complement factor H (CFH), age-related maculopathy susceptibility 2 (ARMS2) and high-temperature requirement factor A1 (HTRA1) have been shown to be associated with AMD in both Japanese and Caucasian patients [4-7]. In addition, complement component 2(C2) and complement factor B (CFB) known as activators of alternative complement cascades are reportedly related to AMD in Caucasians [8]. Both were reported to be protective genes against AMD development [9,10]. Genetic studies of PCV have found no association between either C2 or CFB and PCV [11,12]. Nakata et al. reported that, in the Japanese population, C2 and CFB are associated with both PCV and typical AMD (tAMD) [13].

Polypoidal choroidal vasculopathy (PCV), characterized by a branching vascular network with polypoidal lesions detectable by indocyanine green angiography (IA) [14], is included among the forms of exudative AMD in Japan [15]. Our group previously reported on subtypes of PCV, and categorized PCV as polypoidal choroidal neovascularization (CNV) and PCV in the narrow sense (also referred to as typical PCV) [16]. In the first type, both feeder and draining vessels are visible on IA and network vessels are numerous. This type is thought to be the representative form of CNV beneath the retinal pigment epithelium. In the second group, neither feeder nor draining vessels are detectable and the number of network vessels is small. This type is thought to represent an abnormality of the choroidal vasculature based on hyaline arteriosclerosis [17]. We also showed that there are differences in these two types classified according to IA and optic coherence tomography findings [18]. Genetically, we demonstrated an association between the ARMS2 gene and these two types of PCV [19]. There was a significant ARMS2 gene difference in case–control studies of polypoidal CNV, but no difference between the typical PCV and control groups. This observation suggests that PCV might be genetically divisible into polypoidal CNV and typical PCV.

The possibility of dividing PCV into two types has been raised by other investigators. Okubo et al. reported that PCV can be divided into two types; the small-short and large-long types, but the clinical features in their report differed from those described by our group [20]. Miki et al. recently advocated dividing PCV into polypoidal lesions with a clear branching vascular network and polypoidal lesions without such a vascular network [21]. After classifying PCV into two types based on IA findings, we conducted ARMS2 and CFH genotyping for our patients. The results were highly consistent with our report showing typical PCV to be unrelated to the ARMS2 gene.

The present study aimed to investigate whether there is an association between the C2 or the CFB gene and any of the subtypes of PCV. To our knowledge, this is the first study to examine associations of the C2 and CFB genes with PCV subtypes.

## Methods

### Participants

Six hundred and seventy-seven patients diagnosed as having AMD at Nihon University Surugadai Hospital in Tokyo were enrolled in this study between 2008 and 2010 (472 men, 205 women; mean age 72.11 years). We then categorized AMD as tAMD, PCV and RAP based on IA and color photograph. (tAMD; 187 men, 63 women, PCV; 266 men, 110 women, RAP;19 men, 32 women)

**Figure 1:**
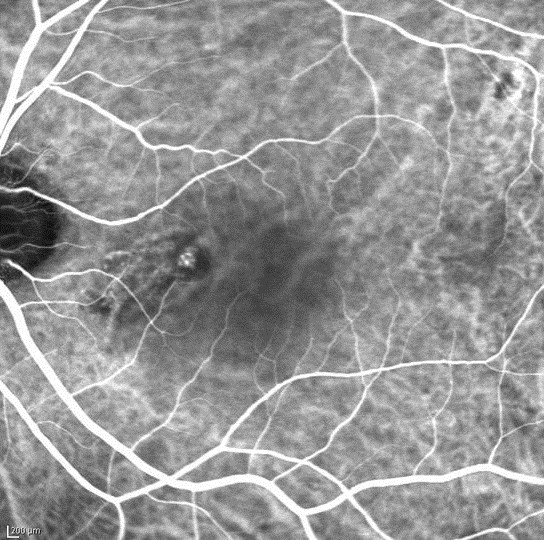
**Typical PCV.** Neither feeder nor draining vessels were visible in the early phase of indocyanine green angiography. The network is composed of a small number of vessels with a polypoidal lesion.

**Figure 2:**
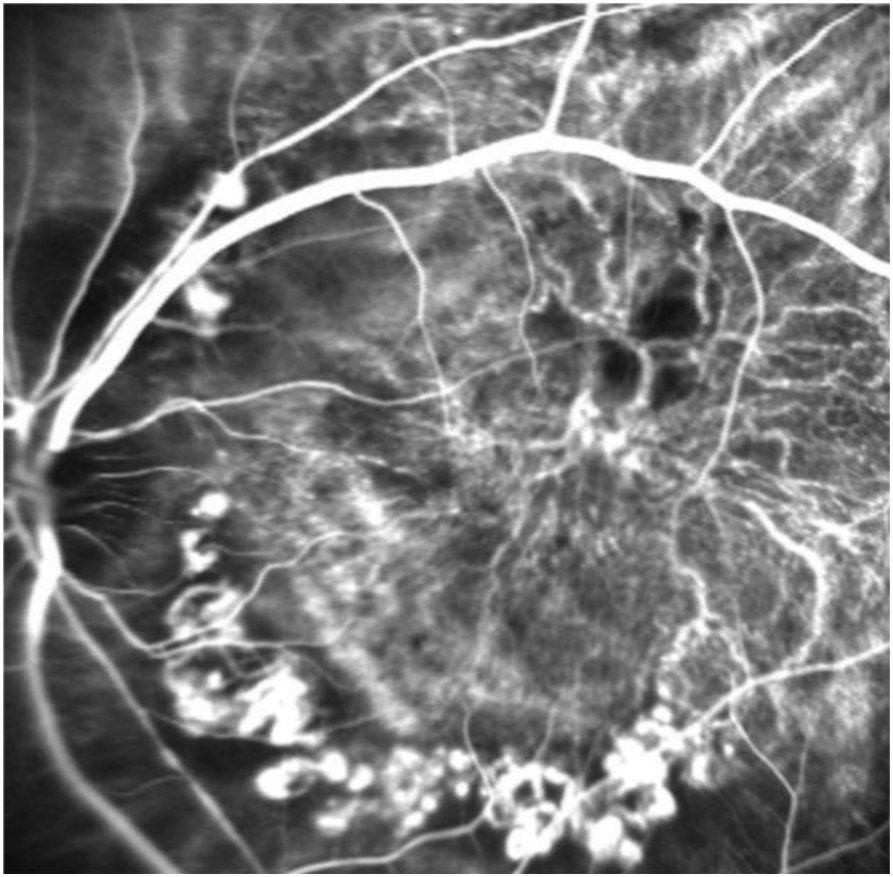
**Polypoidal CNV.** Both feeder and draining vessels were observed in the early phase of indocyanine green angiography. Large numbers of network vessels were seen to be fluorescing in an umbrella-like configuration. Several of the polypoidal lesions were dilatations of marginal tortuous vessels.

Furthermore, we also classified PCV patients into groups with two different types of PCV, polypoidal CNV and typical PCV. Two hundred and eighty-two (195 men, 87 women; mean age 70.0 ± 8.8 years) out of 376 patients were enrolled after classification based on whether or not both feeder and draining vessels were seen on IA (Figures 1 and 2). Due to unclear IA findings, we could not classify the remaining 94 patients. Eighty-four patients were diagnosed with polypoidal CNV, 198 with typical PCV. Polyp numbers and greatest linear dimension (GLD) were determined by IA at the first visit.

Information on hypertension, diabetes mellitus and smoking was obtained from medical histories collected for each patient. Smokers were defined as current or former smokers, whereas non-smokers were defined as subjects with no previous or current smoking history.

In total, 274 subjects free of AMD (110 men, 164 women; mean age 72.9 ± 7.4 years) served as controls. There were no remarkable findings on fundus examinations of the controls. Informed consent was obtained from each individual as per the protocol approved by the Human Studies Committee of Nihon University. This investigation was performed according to the guidelines of the Declaration of Helsinki.

### Genotyping

DNA was extracted from peripheral blood leukocytes by the phenol and chloroform extraction method [22,23]. Genotyping was performed using the TaqMan® SNP Genotyping Assay (Applied Biosystems Inc. Foster City, CA, USA). TaqMan® SNP Genotyping Assays were performed using the Taq amplification method [22,23].

We targeted C2 rs547154(IVS10), and CFB rs541862, rs2072633(IVS17) and rs4151667(H9L), all of which were identified as having positive associations with AMD in prior studies [11,13].

Plates were read on the SDS 7700 instrument with the end-point analysis mode of the SDS version 1.6.3 software package (Applied Biosystems). Genotypes were determined visually based on the dye-component fluorescent emission data depicted in the X-Y scatter-plot of the SDS software. Genotypes were also determined automatically by the signal processing algorithms of the software [22,23].

### Statistical analysis

Data are shown as means ± SD. Differences between the PCV subtype and control groups were assessed by analysis of variance (ANOVA) followed by Fisher’s protected least significant difference test. Hardy-Weinberg equilibrium was assessed by chi-squared analysis. The overall distribution of alleles was analyzed using 2 × 2 contingency tables. The distribution of the genotypes between patient groups and controls was tested using a 2-sided Fisher’s exact test and multiple logistic regression analysis. After Bonferroni correction, statistical significance was set at p < 0.0125.

Based on the genotype data of the genetic variations, linkage disequilibrium (LD) analyses and a haplotype-based case–control study were carried out using the expectation maximization algorithm with the SNPAlyze software program ver3.2 (Dynacom, Yokohama, Japan). |*D*’| values > 0.5 were used to assign SNP locations to one haplotype block. The frequency distribution of occurrence of the haplotypes was calculated by *χ*^2^ analyses.

## Results

The clinical features of AMD patients and the control group are shown in Table 1. Distributions of genotypes and alleles are shown in Table 2. Four variants were in Hardy-Weinberg equilibrium in the control group (data not shown, p > 0.05). There were significant differences in PCV the allele distributions of rs547154 (*C2* gene) and rs2072633 (*CFB* gene) between the PCV group and the controls. The RAP allele distribution of rs2072633 differed significantly between the RAP group and the controls. The tAMD group showed no difference from the controls.

**Table 1:** Characteristics of study participants

	**Case**								**Control**
	**Total AMD**	**P vs. control**	**Typical AMD**	**P vs. control**	**PCV**	**P vs. control**	**RAP**	**P vs. control**	
Subjects, n	677		250		376		51		274
Male/female	472/205	<0.0001*	187/63	<0.0001*	266/110	<0.0001*	19/32	0.757	110/164
Age	72.1(±8.7)	0.157	73.6(±7.5)	0.289	70.0(±8.9)	<0.0001*	80.9(±6.8)	<0.0001*	72.9(±7.4)
HT	39%	0.308	41%	0.658	38%	0.226	41%	0.878	43%
DM	11%	<0.0001*	14%	0.079	9%	0.081	6%	0.016*	20%
Smoker	35%	<0.0001*	37%	<0.0001*	36%	<0.0001*	16%	84%	18%

p-values reflect comparisons between each of the case groups and the control group, calculated using Fisher’s exact test.

*p < 0.05.

**Table 2:** Genotype and allele distributions in AMD patients and control group

				**Total**													
				**Total AMD patients**			**tAMD**			**PCV**			**RAP**			**Control**	
				**n**	**%**	**p-value**	**n**	**%**	**p-value**	**n**	**%**	**p-value**	**n**	**%**	**p-value**	**n**	**%**
rs547154	Genotype		G/G	601	88.8%	0.024	221	88.4%	0.192	335	89.1%	0.053	45	88.2%	0.561	229	83.6%
			T/G	74	10.9%		28	11.2%		40	10.6%		6	11.8%		41	15.0%
			T/T	2	0.3%		1	0.4%		1	0.3%		0	0.0%		4	1.5%
		Dominant model	G/G	601	88.8%	0.032	221	88.4%	0.132	335	89.1%	0.046	45	88.2%	0.530	229	83.6%
			TG + TT	76	11.2%		29	11.6%		41	10.9%		6	11.8%		45	16.4%
		Recessive model	TT	2	0.3%	0.061	1	0.4%	0.375	1	0.3%	0.168	0	0.0%	0.385	4	1.5%
	Allele		G	1276	94.2%	0.004*	470	94.0%	0.026	710	94.4%	0.005*	96	94.1%	0.260	499	91.1%
			T	78	5.8%		30	6.0%		42	5.6%		6	5.9%		49	8.9%
rs541862	Genotype		T/T	600	88.6%	0.054	221	88.4%	0.246	335	89.1%	0.078	44	86.3%	0.715	229	83.6%
			T/C	75	11.1%		28	11.2%		40	10.6%		7	13.7%		42	15.3%
			C/C	2	0.3%		1	0.4%		1	0.3%		0	00.0%		3	1.1%
		Dominant model	T/T	600	88.6%	0.038	221	88.4%	0.132	335	89.1%	0.046	44	86.3%	0.835	229	83.6%
			TC + CC	77	11.4%		29	11.6%		41	10.9%		7	13.7%		45	16.4%
		Recessive model	C/C	2	0.3%	0.147	1	0.4%	0.625	1	0.3%	0.315	00.0%	0.453	3	1.1%	
			TC + TT	675	99.7%		249	99.6%		375	99.7%	51		100.0%		271	98.9%
	Allele		T	1275	94.2%	0.025	470	94.0%	0.099	710	94.4%	0.027	95	93.1%	0.698	500	91.2%
			C	79	5.8%		30	6.0%		42	5.6%		7	6.9%		48	8.8%
rs2072633	rs2072633		G/G	115	17.0%	0.0024*	48	19.2%	0.120	61	16.2%	0.0026*	6	11.8%	0.048	69	25.2%
			G/A	323	47.7%		120	48.0%		178	47.3%		25	49.0%		134	48.9%
			A/A	239	35.3%		82	32.8%		137	36.4%		20	39.2%		71	25.9%
		Dominant model	G/G	115	17.0%	0.0038*	48	19.2%	0.101	61	16.2%	0.0048*	6	11.8%	0.037	69	25.2%
			GA + AA	562	83.0%		202	80.8%		315	83.8%		45	88.2%		205	74.8%
		Recessive model	A/A	239	35.3%	0.0051*	82	32.8%	0.083	137	36.4%	0.0045*	20	39.2%	0.052	71	25.9%
			GA + GG	438	64.7%		168	67.2%		239	63.6%		31	60.8%		203	74.1%
	Allele		G	553	40.8%	0.0005*	216	43.2%	0.037	300	39.9%	0.0005*	37	36.3%	0.013	272	49.6%
			A	801	59.2%		284	56.8%		452	60.1%		65	63.7%		276	50.4%
rs4151667	Genotype		T/T	653	96.5%	0.386	241	96.4%	0.514	363	96.5%	0.412	49	96.1%	0.797	261	95.3%
			A/T	24	3.5%		9	3.6%		13	3.5%		2	3.9%		13	4.7%
			A/A	0	0.0%		0	0.0%		0	0.0%		0	0.0%		0	0.0%
		Dominant mode	T/T	653	96.5%	0.459	241	96.4%	0.664	363	96.5%	0.424	49	96.1%	0.797	261	95.3%
			AT + AA	24	3.5%		9	3.6%		13	3.5%		2	3.9%		13	4.7%
		Recessive model	A/A	0	0.0%	-	0	0.0%	-	0	0.0%	-	0	0.0%	-	0	0.0%
			AT + TT	677	100.0%		250	100.0%		376	100.0%		51	100.0%		274	100.0%
	Allele		T	1330	98.2%	0.461	491	98.2%	0.667	739	98.3%	0.429	100	98.0%	0.779	535	97.6%
			A	24	1.8%		9	1.8%		13	1.7%		2	2.0%		13	2.4%

AMD; age related macular degeneration tAMD; typical age related macular degeneration PCV; polypoidal choroidal vasculopathy RAP; retinal angiomatous proliferation.

*P*- values are for the comparison between cases and controls.

*p*- values for genotypes were calculated by Fisher’s exact test. (after Bonferroni correction *p < 0.0125).

The results of logistic regression analysis, with adjustment for confounding factors, including age, gender and risk factors, are shown in Table 3. This analysis was performed for the dominant or recessive genotype models showing significant results, as presented in Table 2. Susceptibility genotypes were those with high frequencies in patient groups in case–control studies. The rs2072633 distribution of the controls differed significantly from those of the tAMD, PCV and RAP groups. After Bonferroni correction, only PCV showed significant difference in this SNP.

**Table 3:** Logistic regression analysis with adjustment for confounding factors

		**Total AMD patients**				**tAMD**				**PCV**				**RAP**			
		***p-value vs. control***	***(Bonferroni correction)***	**OR**	**95% CI**	***p-value vs. control***	***(Bonferroni correction)***	**OR**	**95%CI**	***p-value vs. control***	***(Bonferroni correction)***	**OR**	**95% CI**	***p-value vs. control***	***(Bonferroni correction)***	**OR**	**95% CI**
rs547154	dominant model	0.044*	0.176	***0.62***	0.39-0.99	0.186	0.744			0.103	0.412			0.626	1.000		
	recessive model	0.051	0.204			0.159	0.636			0.105	0.420			-			
rs541862	dominant model	0.049*	0.196	***0.63***	0.39-0.99	0.169	0.676			0.119	0.476			0.877	1.000		
	recessive model	0.133	0.532			0.232	0.928			0.213	0.852			0.996	1.000		
rs2072633	dominant model	0.0003*	0.001**	***0.49***	0.33-0.73	0.042*	0.168	***0.59***	0.36-0.99	0.001*	0.004**			0.048*	0.192	***0.35***	0.13-0.99
	recessive model	0.031*	0.124	***0.68***	0.48-0.97	0.312	1			0.010*	0.04**	***0.46***	0.29-0.74	0.423	1.000		
rs4151667	dominant model	0.273	1			0.952	1			0.270	1	***0.59***	0.40-0.88	0.769	1.000		
	recessive model	-				-				-				-			

Logistic regression analysis was performed for each genotype with adjustment for confounding factors (age, gender, hypertension, diabetes mellitus and smoking).

PCV; polypoidal choroidal vasculopathy.

OR; odds ratios CI; confidence intervals.

p-values are for the comparisons between cases and controls.

p-values for genotypes were calculated using Fisher’s exact test. *p < 0.05B.

Bonferroni correction was performed for each of the genotypes. **p < 0.05.

Blanks indicate that there were no siginificant differences.

The clinical features of PCV patients and the control group are shown in Table 4. There were significant differences in polyp numbers and GLD, both of which were greater in polypoidal CNV group.

**Table 4:** Characteristics of PCV participants

	**Case**							**Control**
	**Total PCV**	**P vs. control**	**Polypoidal CNV**	**P vs. control**	**P vs. typical PCV**	**Typical PCV**	**P vs. control**	
Subjects, n	282		84			198		274
Male/female	195/87	<0.0001*	63/21	<0.0001*	0.205	132/66	<0.0001*	110/164
Age(±SD)	70.0(±8.7)	<0.0001*	68.8(±8.9)	<0.0001*	0.130	70.5(±8.7)	<0.0001*	72.9(±7.4)
Hypertension	39%	0.390	38%	0.45	0.792	40%	0.509	43%
Diabetes	9%	<0.0001*	10%	0.032*	0.649	8%	<0.0001*	20%
Smoking	33%	<0.0001*	37%	<0.0001*	0.406	31%	0.001*	18%
Number of polyps	-		4.17		<0.0001*	1.95		-
GLD, mm	-		3.78		<0.0001*	2.78		-

p-values reflect comparisons between each of the case groups and the control group, calculated using Fisher’s exact test *p < 0.05.

PCV; polypoidal choroidal vasculopathy CNV; choroidal neovascularization GLD; greatest linear dimension SD; standard deviation.

Distributions of genotypes and alleles of the four variants are shown in Table 5. Four variants were in Hardy-Weinberg equilibrium in the control group (data not shown, p > 0.05). There were significant differences in all genotype models and allele distributions of rs547154 (*C2* gene), rs541862 and rs2072633 (CFB gene), but not rs4151667, between the polypoidal CNV group and the controls. However, there were no significant differences in any genotype model or allele distribution for any of the SNPs between the typical PCV and control groups.

**Table 5:** Genotype and allele distributions in PCV patients and control group

			**Total PCV patients**			**Polypoidal CNV**			**Typical PCV**			**Control**	
			**Number**	**%**	**p-value**	**Number**	**%**	**p-value**	**Number**	**%**	**p-value**	**Number**	**%**
rs547154	Genotype	G/G	255	90.4%	80	95%	175	88%	229	84%			
		T/G	26	9.2%	0.0400	4	5%	0.023	22	11%	0.276	41	15%
		T/T	1	0.4%		0	0%		1	1%		4	1%
	Dominant model	G/G	255	90.4%	0.016	80	95%	0.007*	175	88%	0.142	229	84%
		TG + TT	27	9.6%		4	5%		23	12%		45	16%
	Recessive model	TT	1	0.4%	0.168	0	0%	0.265	1	1%	0.317	4	1%
		TG + GG	281	99.6%		84	100%		197	99%		270	99%
	Allele	G	536	95.0%	0.009*	164	98%	0.004*	372	94%	0.110	499	91%
		T	28	5.0%		4	2%		24	6%		49	9%
rs541862	Genotype	T/T	255	90.4%		80	95%		175	88%		229	84%
		T/C	26	9.2%	0.049	4	5%	0.023	22	11%	0.318	42	15%
		C/C	1	0.4%		0	0%		1	1%		3	1%
	Dominant model	T/T	255	90.4%	0.016	80	95%	0.007*	175	88%	0.142	229	84%
		TC + CC	27	9.6%		4	5%		23	12%		45	16%
	Recessive model	C/C	1	0.4%	0.302	0	0%	0.336	1	1%	0.490	3	1%
		TC + TT	281	99.6%		84	100%		197	99%		271	99%
	Allele	T	536	95.0%	0.013	164	98%	0.004*	372	94%	0.137	500	91%
		C	28	5.0%		4	2%		24	6%		48	9%
rs2072633	Genotype	G/G	50	17.7%		13	15%		37	19%		69	25%
		G/A	131	46.5%	0.017	34	40%	0.005*	97	49%	0.149	134	49%
		A/A	101	35.8%		37	44%		64	32%		71	26%
	Dominant model	G/G	50	17.7%	0.032	13	15%	0.064	37	19%	0.095	69	25%
		GA + AA	232	82.3%		71	85%		161	81%		205	75%
	Recessive model	A/A	101	35.8%	0.012*	37	44%	0.002*	64	32%	0.128	71	26%
		GA + GG	181	64.2%		47	56%		134	68%		203	74%
	Allele	G	231	41.0%	0.004*	60	36%	0.002*	171	43%	0.055	272	50%
		A	333	59.0%		108	64%		225	57%		276	50%
rs4151667	Genotype	T/T	273	96.8%		81	96%		192	97%		261	95%
		A/T	9	3.2%	0.348	3	4%	0.649	6	3%	0.350	13	5%
		A/A	0	0.0%		0	0%		0	0%		0	0%
	Dominant model	T/T	273	96.8%	0.348	81	96%	0.649	192	97%	0.350	261	95%
		AT + AA	9	3.2%		3	4%		6	3%		13	5%
	Recessive model	A/A	0	0.0%	-	0	0%	-	0	0%	-	0	0%
		AT + TT	282	100.0%		84	100%		198	100%		274	100%
	Allele	T	555	98.4%	0.394	165	98%	0.775	390	98%	0.482	535	98%
		A	9	1.6%		3	2%		6	2%		13	2%

PCV; polypoidal choroidal vasculopathy CNV; choroidal neovascularization.

*p*- values are for the comparison between cases and controls.

*p*- values for genotypes were calculated by Fisher’s exact test. (after Bonferroni correction *p < 0.0125).

The results of logistic regression analysis, with adjustment for confounding factors, including age, gender and risk factors, are shown in Tables 6 and 7. This analysis was performed for the dominant or recessive genotype models showing significant results, as presented in Table 5. Susceptibility genotypes were those with high frequencies in patient groups in case–control studies. The distributions of rs541862, rs547154 and rs2072633 differed significantly between the controls and the polypoidal CNV group. After Bonferroni correction, the distribution of rs2072633 remained significant only for polypoidal CNV, i.e. not for typical PCV. Logistic regression analysis was also performed to compare the polypoidal CNV and typical PCV groups. The only significant difference, after adjusting for confounding factors such as polyp numbers and GLD, was in rs2072633. After Bonferroni correction, no significant difference remained.

**Table 6:** Logistic regression analysis between cases and controls

		**Total PCV patients**				**Polypoidal CNV**				**Typical PCV**			
		***p-value vs. control***	***(Bonferroni correction)***	**OR**	**95% CI**	***p-value vs. Control***	***(Bonferroni correction)***	***OR***	**95% CI**	***p-value vs. Control***	***(Bonferroni correction)***	***OR***	**95% CI**
rs547154	dominant model	0.018*	0.072	0.48	0.26-0.89	0.014*	0.056	***0.22***	0.05-0.86	0.139	0.556		
	recessive model	0.217	0.868			0.097	0.388			0.409	1.000		
rs541862	dominant model	0.023*	0.092	0.49	0.26-0.91	0.015*	0.060	***0.22***	0.05-0.87	0.162	0.648		
	recessive model	0.417	1			0.131	0.524			0.646	1.000		
rs2072633	dominant model	0.012*	0.048**	0.52	0.32-0.87	0.104	0.416			0.037*	0.148	***0.55***	0.32-0.96
	recessive model	0.035*	0.140	0.63	0.41-0.96	0.009*	0.036**	***0.40***	0.20-0.79	0.199	0.796		
rs4151667	dominant model	0.326	1			0.984	1			0.211	0.844		
	recessive model	-	-			-	-			-			

Logistic regression analysis was performed for each genotype with adjustment for confounding factors (age, gender, hypertension, diabetes mellitus and smoking).

PCV; polypoidal choroidal vasculopathy.

OR; odds ratios CI; confidence intervals.

*p*-values are for comparisons between cases and controls.

*p*-values for genotypes were calculated using Fisher’s exact test. *p < 0.05.

Bonferroni correction was performed for each of the genotypes. **p < 0.05.

Blanks indicate that there were no siginificant differences.

**Table 7:** Logistic regression analysis between polypoidal CNV and typical PCV

		**Polypoidal CNV**			
		***p-value vs. typical PCV***	***(Bonferroni correction)***	**OR**	**95%CI**
rs547154	dominant model	0.073	0.292		
	recessive model	0.392	1		
rs541862	dominant model	0.073	0.292		
	recessive model	0.392	1		
rs2072633	dominant model	0.720	1		
	recessive model	0.038*	0.152	***2.09***	1.04-4.22
rs4151667	dominant model	0.915	1		
	recessive model	-	-		

Logistic regression analysis was performed for each genotype with adjustment for confounding factors (age, gender, hypertension, diabetes mellitus and smoking).

OR; odds ratios CI; confidence intervals GLD; greatest linear dimension.

*p*-values are for the comparisons between polypoidal CNV and typical PCV.

*p*-values for genotypes were calculated using Fisher’s exact test. *p < 0.05.

Bonferroni correction were performed for each genotypes. p < 0.05.

Blanks indicate that there were no siginificant differences.

LD was assessed for three SNPs in CFB, and the distribution of estimated haplotype frequencies is shown in Tables 8 and 9. The T-A-T(rs541862-rs2072633-rs4151667) and C-G-T haplotypes both showed strong associations in the polypoidal CNV, typical PCV and control groups. Furthermore, the T-A-A haplotype differed significantly between polypoidal CNV and typical PCV.

**Table 8:** Linkage disequilibrium map through 3 SNPs in CFB gene

	**rs541862**	**rs2072633**	**rs4151667**
rs541862	-	0.929	0.278
rs2072633	-0.040	-	1
rs4151667	-0.001	0.012	-

The upper right shows the D’-value, the lower left the D-value.

**Table 9:** Haplotype association analysis in cases and controls

**Polypoidal CNV vs. control**						
**Haplotypes**			**%**			
**rs541862**	**rs2072633**	**rs4151667**	**Polypoidal CNV**	**Control**	**Chi-Squ**	**p-value**
T	A	T	63%	42%	22.177	<0.0001*
C	A	T	0%	9%	14.9366	0.0001*
T	G	T	35%	47%	7.9166	0.0049*
C	G	T	2%	0%	13.5581	0.0002*
T	G	A	0%	2%	3.9293	0.0475*
**Typical PCV vs. control**						
**Haplotypes**			**%**			
**rs541862**	**rs2072633**	**rs4151667**	**Typical PCV**	**Control**	**Chi-Square**	**p-value**
T	A	T	57%	42%	20.5614	<0.0001*
C	A	T	0%	9%	34.8144	<0.0001*
T	G	T	37%	47%	9.3704	0.0022*
C	G	T	6%	0%	33.5324	<0.0001*
**Polypoidal CNV vs. typical PCV**						
**Haplotypes**			**%**			
**rs541862**	**rs2072633**	**rs4151667**	**Typical PCV**	**Polypoidal CNV**	**Chi-Squ**	**p-value**
T	A	T	57%	63%	1.5687	0.2104
C	A	T	0%	0%	0	1
T	G	T	36%	33%	0.3302	0.5656
C	G	T	6%	2%	3.0395	0.0813
T	A	A	0%	2%	7.1092	0.0077*
C	A	A	0%	0%	0	1
T	G	A	1%	0%	2.1402	0.1435
C	G	A	0%	0%	0	1

*p-value > 0.05 calculated by chi-square analysis.

## Discussion

ARMS2 genes, especially the rs10490924 of CFH and rs1061170, are both known as PCV susceptibility genes [24,25]. On the other hand, our group previously reported that typical PCV did not correlate significantly with rs10490924 [19]. This result raised the possibility of two distinct genetic types of PCV. In the present study, the C2 gene and the CFB gene were also found to be associated with polypoidal CNV, in terms of both genotypes and allele distributions. No associations with typical PCV were detected. These results indicate the C2 and CFB genes to also be associated with PCV subtypes. Our group recently reported typical PCV to have the features of abnormal choroidal vessels and that polypoidal CNV also has features of neovascularization. The differences between tAMD and polypoidal CNV were that the latter had polypoidal lesion detectable by IA, while tAMD had no polypoidal lesion. Furthermore, polypoidal CNV is characterized by a larger GLD and more polyps than typical PCV [18]. As polypoidal CNV has neovascularization features, the ARMS2 gene might be highly associated with neovascularization. Though there are reports describing rs4151667 as being associated with AMD, the minor allele homozygous frequency was very low in all of these reports [9,10]. In this study, the minor allele homozygous frequency of rs4151667 was zero, such that there was no difference between cases and controls.

Nakata et al. reported the C2 (rs547154) and CFB (rs541862) genes to be significantly associated with both tAMD and PCV in the Japanese population [13]. Nevertheless, rs2072633 (CFB gene) and rs4151672 (CFB gene) showed no correlations with either tAMD or PCV. In the present study, we showed rs2072633 to be significantly associated with PCV. This result indicates the CFB genes to be associated with PCV. Before Bonferroni correction, tAMD was also associated with rs547154 and rs2072633. We previously reported that polypoidal CNV resembles tAMD, while typical PCV clearly differs from CNV. Though not significant after Bonferroni correction, given the prior reports dividing PCV into two types, we can reasonably speculate that the C2 and CFB genes might be related to tAMD and polypoidal CNV but not to typical PCV. The present C2 and CFB gene results also are not inconsistent with this possibility. Since typical PCV was not associated with any of the SNPs tested, we can also speculate that typical PCV might differ genetically from AMD.

C2 and CFB functioned as activators of the complement cascade. CFB is localized to the choroidal vasculature and Bruch’s membrane [26]. Smailhodzic et al. reported AMD patients to show increased alternative pathway activation and elevated CFB levels [27]. Scholl et al. also showed plasma CFB to be significantly elevated in AMD patients [28]. For these reasons, AMD might be related to CFB.

Recently, Liu et al. reported the C2-CFB-RDBP-SKIV2L region of SNPs to be associated only with tAMD, not with PCV. They concluded that the mechanisms underlying the development of tAMD and PCV might be different [29]. Nakashizuka et al. reported histopathological characteristics of PCV [17]. In their report, areas of PCV showed little fibrosis or granulation as compared to those with CNV. This might indicate that typical PCV involves less inflammation than CNV. Since polypoidal CNV has AMD features, C2 and CFB might be related only to polypoidal CNV.

The results presented in Table 8 show that three of the SNPs in CFB were in LD block. Haplotypes T-A-T and T-G-T differed significantly between the PCV and control groups. Furthermore, T-A-T would confer a risk for PCV, while T-G-T would be protective against PCV development. We could reasonably draw the same conclusion for haplotypes C-A-T and C-G-T. These results indicate that rs2072633 might be one of the key SNPs favoring PCV development.

There has been controversy regarding the division of PCV into two subtypes. Tsujikawa et al. reported that if there is risk associated with being homozygous for the ARMS2 gene, it would be the larger GLD in PCV [30]. Their report described two types of PCV, with larger GLD and smaller GLD. The aforementioned report by Miki and colleagues presented results very similar to ours, indicating the ARMS2 gene to have no association with typical PCV [21]. These two reports also support the assumption that the ARMS2 gene is unrelated to PCV [17,18]. While IA findings of polypoidal CNV appeared to be consistent with CNV, the histopathological and IA features of typical PCV showed choroidal vasculature abnormalities. These observations suggested polypoidal CNV to be genetically and histopathologically close to tAMD, a representative form of CNV. Furthermore, typical PCV showed no association with CNV.

The small sample size with only one genotype is the major limitation of this study. Further study is clearly needed.

## Conclusion

The present study is the first to examine the associations between variants in the C2 and CFB genes and PCV subtypes. We found the C2 and CFB genes to possibly be genetic markers for polypoidal CNV. Furthermore, these variants showed no associations with typical PCV. These results suggest polypoidal CNV to have a genetic background different from that of typical PCV. Further studies are needed to examine the effects of various treatments on PCV subtypes.

## Abbreviations

PCV: Polypoidal choroidal vasculopathy; CNV: Choroidal neovascularization; C2: Complement component 2; CFB: Complement factor B; AMD: Age-related macular degeneration; CFH: Complement factor H; ARMS2: Age-related maculopathy susceptibility 2; HTRA1: High-temperature requirement factor A1; tAMD: Typical AMD; IA: Indocyanine green angiography; GLD: Greatest linear dimension.

## Competing interests

The authors have no competing interests to declare.

## Authors’ contributions

KT, TN and MY participated in the design of this study. KT and NS participated in the laboratory work. RM and AK were responsible for participants’ enrollment. KT performed the statistical analysis and wrote the draft manuscript. All authors read and approved the final manuscript.

## Pre-publication history

The pre-publication history for this paper can be accessed here:

http://www.biomedcentral.com/1471-2415/14/83/prepub
